# Data Mining Technology Enabled Anti Retroviral Therapy (ART) for HIV Positive Patients in Gondar University Hospital, Ethiopia

**DOI:** 10.6026/97320630015790

**Published:** 2019-12-08

**Authors:** Tamrat Delessa Chala

**Affiliations:** 1 Department of Computer Science, College of Computing and Informatics, Haramaya University, Ethiopia

**Keywords:** Antiretroviral therapy (ART), KDD methodology, J48 pruned decision tree, ART Starter, WEKA 3.7

## Abstract

It is of interest to discuss the feasibility for data mining technology enabled antiretroviral therapy (ART) for HIV positive patients at the University of Gondar Specialized Teaching Hospital, Ethiopia. The Knowledge Discovery in Databases (KDD), which is in an iterative process where evaluation measures enhanced, is used to prepare the data set for ART in HIV positive patients. A decision tree J48 model that is the implementation of algorithm ID3 (Iterative Dichotomiser 3) developed by the WEKA project team is used in this study. The J48 model was built with pruned and without pruned parameters by selecting two different test modes for 10-fold cross validation with percentage split. Results using J48 pruned decision tree with 10-fold cross validation produces 80.5% prediction precision for ART starter prognosis enabled treatment in clinical settings.

## Background

Ethiopia is among the countries most heavily affected by widespread HIV infection. The first evidence of HIV epidemic in Ethiopia was detected in 1984 [1]. Gondar University Hospital in Ethiopia is a tertiary referral teaching hospital with 350 beds giving service to more than 5 million people living in the vicinity areas. The hospital started to provide HIV/AIDS related services in 2003 with limited scope for shortage of trained manpower and fee based Antiretroviral (ARV) drugs. The hospital scaled up its HIV/AIDS related care with the introduction of free ART service in 2005. Currently, the hospital is fully staffed with trained HIV/AIDS health care providers and is supported by a PEPFAR funded international Non-Governmental Organization (NGO), International Training and Education Center for Health (I-TECH), in the area of capacity building with technical and financial support. According to Gondar University Hospital June 2010 hospital HIV/AIDS report, there has been a total of 8377 patients enrolled to the HIV care program, out of which 5566 patients started ART. About 858 patients have been transferred out (TO) to other ART centers and 397 patients were transferred in (TI) to the hospital. There are a cumulative 855 patients who were dropped out and 152 lost from follow-up patients making the overall active patients on ART to 3370 [2].

HIV care is currently periodic acute care with the exception of TB treatment. Therefore, establishing good chronic HIV care including ART requires forming and preparing clinical teams. Those teams provide continuity of HIV care to enable the patients with care and support. Patients are linked with Antiretroviral Therapy (ART) office in University of Gondar Comprehensive Specialized Hospital and large volume of data with many attributes is thus collected. Sometimes patients refuse to be linked with the ART office for care. It should be noted that the database at the University of Gondar Hospital has its own Database on ART care is inadequate to make decision for ART care warranting the addition of more data. A study by Nebiyu Mekonnes et al. on incident and predictors of loss to follow-up among HIV infected adults after initiation of first line ART at University of Gondar Comprehensive Specialized Hospital showed a loss of HIV positive patients ART services at 12.26 per 100 persons per year [3]. Elias Lemuye (2011) applied classification and association rules using J48 and ID3 to investigate HIV status predictive modelling to support the scaling up of HIV testing in Addis Ababa. Cross-industry standard process for data mining (CRISP-DM) is used to show the use of predictive modelling for HIV status data [4]. Vararuk et al. (2008) reported the management of HIV/AIDS using 250,000 records from HIV/AIDS patients in Thailand using the IBM's Intelligent Miner with a data mining technique employing clustering based on common characteristics and errors in the data [5]. Identification of associated symptoms using data gathered by medical practitioner is also reported [6]. Therefore, it is of interest to compile a comprehensive report on the statistics of HIV positive patients with and without ART service to help in the prognosis of the service using data mining techniques (applying rule based models) using known data in the literature.

## Methodology

Knowledge discovery in data bases (KDD) used for data mining has five (5) stages such as: selection, pre-processing, transformation, data mining and interpretation [7].

## Selection:

This stage consists on creating a target dataset, or focusing on a subset of variables or data samples, on which discovery is to be performed.

## Pre-processing:

This stage consists on the target data cleaning and pre processing in order to obtain consistent data. Here we also try to eliminate noise that is present in the data. Noise can be defined as some form of error within the data. Some of the tools used here can be used for filling missing values and elimination of duplicates in the database.

## Transformation:

This stage consists on the transformation of the data using dimensionality reduction or transformation methods. Usually there are cases where there are a high number of attributes in the database for a particular case. With the reduction of dimensionality we increase the efficiency of the data-mining step with respect to the accuracy and time utilization.

## Data mining:

The data-mining step is the major stage in data KDD. This is when the cleaned and preprocessed data is sent into the intelligent algorithms for classification, clustering, similarity search within the data, and so on. Here we chose the algorithms that are suitable for discovering patterns in the data. Some of the algorithms provide better accuracy in terms of knowledge discovery than others. Thus selecting the right algorithms can be crucial at this point [7].

## Interpretation/evaluation:

In this stage the mined data is presented to the end user in a Human view able format. This involves data visualization, which the user interprets and understands the discovered knowledge obtained by the algorithms [7]. The secondary data was collected from University of Gondar Hospital (UoGH) ART databases. UoGH has its own database to store their patients' details for long time; specifically the ART service provider office also has its own database. The data clerks enter the patients' records and generate reports for different purpose. So, the patient data was collected that contains fields Card Number, Registration Date, Age, Sex, ART Stage, Functional Status, Eligible Date and Patient details such as: Occupation, Religion, Education Level, Marital status, Clinical stage, CD4 check, year and Start ART. Finally, selected attributes for this research were: year, sex, age, ART stage, Functional status, marital status, occupation, clinical stage, CD4 check and start ART based on expert recommendation.

## Experimentation and analysis of results:

The main objective of this study is to discover ART service data to distinguish regular patterns in order to enable data mining technology for HIV Positive patients care with ART care service. The advantage of experiments in classification is to discover model that is able to predict the determinant factors of ART service for HIV positive patients. In order to train the classifiers 1002 data are used for training and testing. To creating the predictive model to determine an ART service for HIV positive patients the selected algorithm was decision tree J48 tree with one parameters that was unpruned where it's parameter type is Boolean (TRUE OR FALSE) and for both J48 decision tree with its parameter two test mode has been used such as 10-fold Cross validation and Percentage split with different split of data training and data testing to compare the difference of two test modes with j48 decision tree and JRIP rules. In this study ten (10) attributes have been selected in this paper namely Year, Sex, Age, ART stage, Functional status, occupation, clinical stage, CD4check and Start ART. From all these attributes the dependent attributes is Start ART. So, the following eight (8) scenarios were used for the experiment.

[1] Type#1: Decision tree J48 pruned tree with test mode 10-fold cross validation

[2] Type#2: Decision tree J48 pruned tree with test mode percentage split to 50% 

[3] Type#3: Decision tree J48unprunedtree with test mode 10-fold cross validation

[4] Type#4: Decision tree J48unprunedtree with test mode percentage split to 50% 

[5] Type#5: Decision tree J48 pruned tree with test mode percentage split by 80% to 20%. 

[6] Type#6: Decision tree J48unprunedtree with test mode percentage split by 80% to 20 

[7] Type#7 rules JRIP rules with test mode 10-fold cross validation.

[8] Type#8 rules JRIP rules with test mode percentage split by 66% to 34%

## Methods of Analysis and Evaluation of the model:

Experiment of Type#1

The result obtained by experiment Type#1 is as shown in (Table 2)

The outputs of series of experiments of classification models are analyzed and evaluated in terms of the details of the confusion matrix of the model as shown in (Table 1). The complexity of the model in terms of the number of trees and leaves are also evaluated.

Therefore from the above sample output shows that the accuracy is 80.5% that means out of 1002 instance 80.5% was correctly classified. The confusion matrix shows that, out of the total 612 actual of Patients that starter of ART service only 544 (88.9%) and the left are 11.1% were misclassified as not to start ART service. And out of the 390 instances only 263 were actually classified as not to start ART service (67.4%) and the rest have been misclassified as to start ART service around (32.6%). From this model, the performance obtained in terms of correctly classifying the patients those starts ART service than those did not start ART service. So in this confusion matrix there are two error types the first type is the patient those misclassified as no starter of ART service that is 11.1% that is serious problem because there is no awareness of ART service for these people. And the second type of error is those patients misclassified as the patients those starting ART service this error happened due to sometimes HIV positive patients enforced by physician ground on some criteria like pregnancy and other reasons.

Data in Table 3 shows that the accuracy performance is 79.0% that means out of 501 instance 79.0% was correctly classified. The confusion matrix shows that, out of the total 304 those Patients that is actual starter of the ART service only 267(87.8%) and the left are 12.2% have been misclassified as not to start ART service. And out of the 197 instance only 129were classified as not to start ART service (65.5%) and the rest were misclassified as to start ART service around (34.5%). From this model researcher conclude that the same with J48 with test mode 10-fold cross validation it is performance is good in terms of correctly classifying the patients those start ART service than those did not start ART service. Again in this confusion matrix also two-error types was happened the first type is the patients those misclassified as no starter of ART service that is 12.2% is serious problem because there awareness is not created about ART service for these people. Here when we compared the first model with 10-fold cross validation and this model with the percentage 50% split the first model means 10-fold cross validation is good than the percentage split to 50%. Because of by 10-fold cross validation test mode the error is only 11.1% which is 12.2% by the percentage split 50% their difference is 1.1%.And the second type of error is those patients misclassified as the patients those starting ART service this error is no this much as the first problem because of sometimes based on the criteria of WHO before the patients start the ART service they can be wait in care of another treatment such as like counseling and sometimes also based on the patients immunity directly they enforced as they have been starting ART service.

In the third scenario that is J48 unpruned tree when the test mode is 10-fold cross validation its sample output shows as depicted in the Table 4 that the accuracy performance is 79.3% that means out of 1002 instance 79.0% is correctly classified. The confusion matrix shows that, out of the total 612 those patients that actual starter of the ART service only 522 (85.3%) and the left are 14.7% were misclassified as not to start ART service. And out of the 390 instance only 273were classified as not to start ART service (70.0%) and the rest are misclassified as to start ART service around (30.0%). From this model we can conclude that the same with J48 pruned tree with test mode 10-fold cross validation it is very good performance in terms of correctly classifying the patients those start ART service than those did not start ART service. Again in this confusion matrix also there is two error types the first type is the patients those misclassified as no starter of ART service that is 14.7% which is serious problem. And the second type of error is those patients misclassified (30.0%) as the patients those starting ART service this error is no this much as the first problem because of sometimes based on the criteria of WHO before the patients start the ART service they can be wait in care of another treatment such as like counselling and another.

In the fourth case (Table 5)of this model that is J48 unpruned tree when the test mode is percentage split 50.0% train, remainder test its sample output have been shown that the accuracy performance is 77.1% that means out of 501 instance 77.1% is correctly classified. The confusion matrix shows that, out of the instance 304 those patients that actual starter of the ART service only250 (82.2%) and the left are 17.8% were misclassified as not to start ART service. And out of the 197 instance only 136 have been classified as not to start ART service (69.0%) and the rest are misclassified as to start ART service around (31.0%). When this model compared with the above three model it less accuracy than them. However, still this model is better performance in terms of correctly classifying the patients those should be start ART service than those not start ART service. Again this model has also high percentage error Type1 than the above three model that is the patients those misclassified as no starter of ART service that is 17.7% which is serious problem because there is no awareness about ART service at all for these people. The second type of error is those patients misclassified (31.0%) as the patients those starting ART service this error is no serious as the first problem because of sometimes based on the criteria of WHO if the condition is not allowed for patients sometimes the physicians enforced them as they immediately start the ART service for patients benefit.

As the result achieved in the (Table 6) the accuracy performance is 78.0% this means out of 200 instance 78.0% is correctly classified and the left was incorrectly classified by 22% out of 200 instances. The confusion matrix shows that, out of the total 118 those Patients that is actual starter of the ART service only 103 (87.3%) and the left are 12.7% were misclassified as not to start ART service. And out of the 82 instances only 53 were classified as not to start ART service (64.6%) and the rest are misclassified as to start ART service around (35.4%). As the result indicated that this model very good performance in terms of correctly classifying the patients those starts ART service than those did not start ART service. On another hand, from this confusion matrix two errors have been analyzed the first error is the patients those should be start ART, but misclassified as no start ART service they are in percent 12.7%. This error is as already described in other models it is severe error because of those patients should begin ART service to live long time in appropriate way which is highly recommended by physicians.

The second type of error is those patients as rules not start ART service, but misclassified as the patients those start ART service in percentage they are around 35.4%. When second type of error compared with the first error the second one is recommended by physicians based on the patients' type for instance if the patients are attacked by TB disease or pregnant and they are recommended as they directly start ART service without any criteria.

As the above sample output (Table 7) shows that the accuracy performance is 77.5% this means out of 200 instance 77.5% is correctly classified and the left was incorrectly classified by 22.5% out of 200 instances. As confusion matrix shows that, out of the total 118 those Patients that is actual starter of the ART service only 100 (84.7%) and the left are 15.3% are misclassified as not to start ART service. And out of the 82 instances only 55 are classified as not to start ART service (67.1%) and others were misclassified as to start ART service around (32.9%). Therefore this model well performed in terms of accuracy because it classifies the patients those start ART service than those did not start ART service. Again from this model also the researcher analysis two types of errors: The first error is which the patients misclassified as not to start ART service rather they should be start ART service which never recommended by physicians in percentage they are around 15.3%. The second error is that the patients misclassified as the patients the starter of ART service that means when the criteria of they start ART service is not fulfill. As the professionals was states this error has not disadvantage because sometimes based on some conditions the physicians/experts can decide as the patients directly start ART service without any pre conditions.

## Comparison and performance:

Based on the above result of each individual model of decision tree model with all of them use same attributes compare and contrast as shown in the Table 8.

Hence if we compared them in terms of accuracy with each other with some extent Type#1 is highly precision than others because of its accuracy is 80.5% and the F-measure for yes is higher than the others F-measures for yes. F-measure is used to balance the precision and recall. And Type#4 is less accuracy than others. In terms of efficiency those take less memory are Type#1, Type#2 and Type#5 are better than Type#3, Type#4 and Type#6.These results have been shown that if the decision tree J48 is with pruned it takes less memory than J48 decision tree without pruned. So this is show that if the parameter is pruned it is good to save memory. On another hand, in terms of time relatively decision tree J48 unpruned is good than decision tree J48 with pruned. Finally from the above models when the percentage split test mode was used the researcher tested by split the data set into different percentage of data training and data test. That is by made 50%-50% for both pruned and without pruned parameter and made 80% data training and 20% data test. In this case with some extent their accuracy result was different from each other. The parameter is pruned and then dataset is split into 50%-50%, which is better than 80%-20% by 1%. If the parameter is without pruned then data to split into 80%-20% is better than 50%-50% by 0 .5%.

Where:

[1] Type#1: Decision tree J48 pruned tree with test mode 10-fold cross validation

[2] Type#2: Decision tree J48 pruned tree with test mode percentage split to 50% 

[3] Type#3: Decision tree J48unpruned tree with test mode 10-fold cross validation

[4] Type#4: Decision tree J48unpruned tree with test mode percentage split to 50% 

[5] Type#5: Decision tree J48pruned tree with test mode percentage split by 80% to 20%

[6] Type#6: Decision tree J48unpruned tree with test mode percentage split by 80% to 20%

## Comparison and performance:

Experiment of Type#7

This is the JRIP rule model build for Determine of ART service for HIV positive patients, so this model is also high precision as the above decision tree J48 pruned tree or unpruned tree in terms of correctly classified data is around 78.9% from 1002 instances and 21.1% misclassified out of instances (Table 9). Its confusion matrix is also almost the same with the above scenarios. Particularly from this model the data mining is discovered the new knowledge based on the above rules.

Rule 1: If CD4 count is greater than or equal to 3 and the registration date is 2008/9 or less than this year and CD4 count is less than 119 then the starting of ART care service is most probably to be predicted as No by ratio (107.0/12.0) from this rule the knowledge discovered is before 2008/9 the awareness of the ART service is less in all our country and in the Gondar town also.

Rule 2: If ART stage is 'EL' or Eligible and year the patients registered is less than or equal to 2008/9 and CD4 count is greater than 126 then the probability of the patient to start ART care service is predicted to No by the ratio (86.0/14.0). From this rule the hidden knowledge discovered is some patients were not interest to start ART care service as the expert is stated it is difficult to predict the reason but many times there is the cultural impact which the patients fear to continue the ART service and they have not confidence to act as the patients those have HIV virus.

Rule 3: If ART stage is 'EL' or Eligible and Functional status is 'B' or Bedridden THEN the Starting of ART service is highly likely to be predicted as No that means not start ART service because of may be the patients can be exposed by another disease no long live or as the experts stated they have not a great chance to live if they patients those HIV positive are Bedridden.

Rule 4: If clinical stage is 1 and 2 and the ART stage is 'IN' or in care THEN the starting of ART service most likely predicted to NO by the ratio (41.0/4.0). From this if the CD4 is not count for the patients the physician decide the continuity of ART service based on the clinical stage that means if the clinical or WHO stage of the patients is 1 or 2 most probably the patient is not start ART service.

Rule 5: If ART stage is 'EL' or Eligible and CD4 result is greater than 9 and less than or equal to 259 and Age greater than 35 THEN the starting of ART service is predicted to NO by the ratio (25.0/8.0). From this rule the hidden knowledge as the expert was stated when the age of patients increased the probability they developed another disease is high because of the disease most patients can be died so they did not start ART service.

This is the final model build used for Determine of ART service for HIV positive patients, so this model is also high precision as seven others models in terms accuracy that means correctly classified data is around 79.5% from 341 instances and 20.5% misclassified out 341 of instances (Table 10). As confusion matrix shows that, out of the total 201 those Patients that is actual starter of the ART service only 175 (87.1%) and the left 12.9% were misclassified as not to start ART service. And out of the 140 instance only 44 are classified as not to start ART service (68.6%) and the rest were misclassified as to start ART service around (31.4%). Hence this model is also very good performance in terms of accuracy because it classifies the patients those start ART service than those did not start ART service. Again from this model also the researcher analysis two types of errors: The first error is which the patients misclassified as not to start ART service rather they should be start ART service which never recommended by physicians in percentage they are around 12.9%. The second error is that the patients misclassified as the patients the starter of ART service that means when the criteria of they start ART service is not fulfill. As the professionals was states this error has not disadvantage because sometimes based on some conditions the physicians/experts can decide as the patients directly start ART service without any pre conditions. And also the rules of this model are the same with the rules of JRIP rules of 10-fold cross validation. When we compared JRIP rules algorithm by two different test modes such as 10-fold cross validation and percentage split 66%-34%. The second JRIP that means JRIP rule with test mode percentage split to 66%-34% is more performance than JRIP rule with 10-fold cross validation by 0.6%. Finally, when we compared the decision tree J48 and JRIP rules with some extent the J48 decision tree is high accuracy than the JRIP rules. And the run time JRIP takes more than J48 decision tree run time. Therefore, J48 decision tree is more effective and efficient than JRIP rules.

Therefore from the above eight (8) models the researcher selected, as the best performance algorithm is the first type that means Type #1: Decision tree J48 pruned tree with test mode 10-fold cross validation is good performance than others in terms of accuracy. Therefore, model 1 is selected as best model and the researcher used this model to drive some relevant classification rules. Some classification rules can be derived from decision tree models, which those rules are not known by expert domain that means they are interesting rule. These rules are described as the following:

[1] CD4 check <=0 and clinical stage >2 and ART stage is EL or 'Eligible' and year <=2008/9 and Functional status is B or 'bedridden' Then NO.

[2] CD4 check <=0 and clinical stage >2 and ART stage is EL or 'Eligible' and year <=2008/9 and Functional status is A or 'Ambulatory' and Age >=31 Then NO.

[3] CD4 Check <=0 and clinical stage <2 and ARTstage is EL or 'Eligible' and Year >=2008/9 and Functional status is W or 'Worker' and clinical stage > 1 Then YES.

[4] CD4 check <=0 and clinical stage >2 and ARTstage is EL and year <=2008/9 and Functional status is W or 'Worker' and Marital status separated and age <=33 Then NO.

[5] CD4 check > 0 and ART stage is EL or 'Eligible' and Year <= 2008/9 then NO.

[6] CD4 check > 0 and ART stage is EL or 'Eligible' and year is >= 2008/9 and CD4 check >=148 and sex is F then No.

[7] CD4 check > 0 and ART stage is EL or 'Eligible' and year >=2008/9 and year <=2009/10 and Age is <= 14 Then Yes.

[8] CD4 check > 0 and ART stage is EL or 'Eligible' and year >=2008/9 and CD4 check <=148 Then NO.

So, the above rules those with some extent different from the criteria the expert domain used to determine the starter of ART service that is interesting rules. The definitions of these rules are:

[1] Rule 1: If CD4 is not count for the patient and the clinical stage greater than two (2) means stage 3 and stage 4 and ART stage is EL or 'eligible' to start ART and the registered year is less than or equal to 2008/9 and Functional status is B or Bedridden THEN starting of ART service is highly likely to be predicted as No that means not start ART service because may be the patients can be exposed by another disease no long live or as the Experts stated they have not a great chance to live if they patients those HIV positive are Bedridden.

[2] Rule 2: If CD4 is not count and clinical stage is greater than two (2) that is stage 3 and 4 and ART stage is eligible to start ART and the year registered is less than or equal 2008/9 and Functional status is A or 'Ambulatory' and age is greater than or equal to 31 THEN starting of ART service is highly likely to be predicted as NO. This rule is different rule which known by expert and this is rule is shows Error 1 which the patient eligible to start ART but they refuge to start ART service.

[3] Rule 3: If CD4 is not count and clinical stage is greater than two (2) that is stage 3 and 4 and ART stage is eligible to start ART service and year greater than or equal 2008/9 and Functional status is W or Worker and clinical stage greater than 1 THEN starting of ART service is highly likely to be predicted as YES. This rule is also unknown with the expert domain or according to the criteria of WHO this rule is not allowed, so it is surprise rule.

[4] Rule 4: If CD4 is not count and clinical stage is greater than two (2) that is stage 3 and 4 and ART stage is eligible to start ART service and year is less than 2009/10 and Functional status is worker and Marital status separated and age is less than equal to 33 THEN starting of ART service is highly likely to be predicted as NO. This rule is also interesting rules because of those person separated and their age is young means less than 33 they are never interest to start ART service, so awareness is necessary for young age peoples about ART service.

[5] Rule 5: If CD4 is count for patients and ART stage is eligible and year is less than 2008/9 THEN starting ART service is highly likely to be predicted as NO. From this rule before 2008/9, the awareness about ART service in the society was very less.

[6] Rule 6: If CD4 is count for patients and ART stage is eligible and year is greater than or equal to 2008/9 and CD4 is greater than or equal to 148 and the sex is female THEN starting of ART service is highly likely to be predicted as NO. From this decision tree also we can select the interesting attributes based on the information gain such as: CD4 check, Clinical Stage, Art stage, Year and functional status are more interest as we observed from the decision tree. Thus, this study finds 80.5% from the given instances actually data were classified correctly as the starter of ART service and only 19.5% misclassified as not to start ART service which is serious problem. So if it compared with others models this model is best in both case that is the actually classified as to start Art is greater than others and the misclassified as not to start ART service is less than other models in percentage. Finally, although we selected the model of Type#1: Decision tree J48 pruned tree with test mode 10-fold cross validation as the best performance, it scored only 80.5% accuracy. The researcher identified the three major challenges that affected the selected model performance: the first one is weka 3.7.7 tool has no smote which allow us to balance dataset, the second is error 1 which is some patients those eligible to begin ART service, but they do not have willing to start ART service and the last one is error 2 this error is which the patients enforced as they have to begin ART service without any precondition.

## Discussion

Numerous investigates have revealed that data mining technology is applicable for ART services of HIV/AIDS positive patients to prognosis enabled handling in clinical settings [4-6].The applicability of data mining technology allowed antiretroviral therapy (ART) for HIV positive patients tested using 1200 records showed 80.5% accuracy. Eight (8) different models were tested.The best performing model is Decision tree J48 pruned which is a tree with a test mode of 10-fold cross-validation for good accuracy.The study conducted on incident and predictors of loss to follow-up among HIV positive patients showed 12.26 score per 100 people per year [3].We also show that out of 1002 records 19.5% was misclassified as they did not begin ART Service due to several reasons like lack of awareness, mobility, started ART service after bedridden conditions, etc.

## Conclusion

HIV/AIDS has claimed the lives of millions and has left behind hundreds of thousands of orphans in Ethiopia. The government of Ethiopia took several steps in preventing further disease spread, and in increasing accessibility to HIV care, treatment and support for persons living with HIV. The accessibility to HIV care, treatment by giving antiretroviral therapy drugs for patients based on the immunity of individual patients is reasonably successful. We used the ART dataset (with pre-processed information on HIV patients for data mining) with 1002 records to select patients for starting ART service. The Knowledge Discovery in Databases (KDD), which is an iterative process where evaluation measures enhanced, is used to prepare the data set for ART in HIV patients. A decision tree J48 model that is the implementation of algorithm ID3 (Iterative Dichotomiser 3) developed by the WEKA project team is used in this study. The J48 model was built with pruned and without pruned parameters by selecting two different test modes for 10-fold cross validation with percentage split. Results using J48 pruned decision tree with 10-fold cross validation produced 80.5% accurate prediction for ART starter towards prediction-enabled treatment in clinical settings.

## Figures and Tables

**Table 1:** Template of Confusion Matric Analysis Method

		Predictive ART determinant		Total
Actual ART determinant		YES	NO	
	YES	TP	FP	TP +FP
	NO	FN	TN	FN +TN
Total		TP+FN	FP + TN	TP +FN+FP+TN

**Table 2:** Summary of Decision tree J48 pruned tree with test mode 10-fold cross validation

Instances	Number of Classified	Accuracy in %	Confusion Matrix		
				YES	NO
Correctly Classified	807	80.54%	YES	544	68
Incorrectly Classified	195	19.46%	NO	127	263
Total of Instances	1002				

**Table 3:** Summary of Decision tree J48 pruned tree with test mode Percentage split to 50%

Instances	Number of Classified	Accuracy in %	Confusion Matrix		
				YES	NO
Correctly Classified	396	79.04%	YES	267	37
Incorrectly Classified	105	20.96%	NO	68	129
Total of Instances	501				

**Table 4:** Summary of Decision tree J48unpruned tree with test mode 10-fold cross validation

Instances	Number of Classified	Accuracy in %	Confusion Matrix		
				YES	NO
Correctly Classified	795	79.34%	YES	522	90
Incorrectly Classified	207	20.66%	NO	117	273
Total of Instances	1002				

**Table 5:** Summary of Decision tree J48unpruned tree with test mode Percentage split to 50%

Instances	Number of Classified	Accuracy in %	Confusion Matrix		
				YES	NO
Correctly Classified	386	77.05%	YES	250	54
Incorrectly Classified	115	22.95%	NO	61	136
Total of Instances	501				

**Table 6:** Summary of Decision tree J48 pruned tree with test mode Percentage split by 80% to 20%.

Instances	Number of Classified	Accuracy in %	Confusion Matrix		
				YES	NO
Correctly Classified	156	78%	YES	103	15
Incorrectly Classified	44	22%	NO	29	53
Total of Instances	200				

**Table 7:** Summary of Decision tree J48unpruned tree with test mode Percentage split by 80% to 20

Instances	Number of Classified	Accuracy in %	Confusion Matrix		
				YES	NO
Correctly Classified	155	77.50%	YES	100	18
Incorrectly Classified	45	22.50%	NO	27	55
Total of Instances	200				

**Table 8:** Summary result of J48 decision tree algorithm

Type	Number of leaves	Size of tree	Time taken	Accuracy	F-measure For YES	F-measure For NO
Type#1	59	91	0.2	80.50%	84.60%	73.00%
Type#2	59	91	0.03	79.00%	83.60%	71.10%
Type#3	166	237	0.02	79.30%	83.50%	72.50%
Type#4	166	237	0.03	77.00%	81.30%	70.30%
Type#5	59	91	0.48	78%	82.40%	70.70%
Type#6	166	337	0.06	77.50%	81.60%	71.00%

**Table 9:** Summary of rules JRIP rules with test mode 10-fold cross validation

Instances	Number of Classified	Accuracy in %	Confusion Matrix		
				YES	NO
Correctly Classified	791	78.94%	YES	539	73
Incorrectly Classified	211	21.06%	NO	138	252
Total of Instances	1002				

**Table 10:** Summary of rules JRIP rules with test mode percentage split by 66% to 34%

Instances	Number of Classified	Accuracy in %	Confusion Matrix		
				YES	NO
Correctly Classified	271	79.47%	YES	175	26
Incorrectly Classified	70	20.53%	NO	44	96
Total of Instances	341				

## References

[1] HIV/AIDS Bureau (2014). Guide for HIV/AIDS Clinical Care, Health Resources and Services Administration.

[2] Amare  B (2011). J Clinic Experiment Ophthalmol.

[3] Mekonnen  N (2019). BMC Res Notes..

[4] E Lemuye (2012). HIV Status Predictive Modeling Using Data Mining Technology:Predicting HIV status.

[5] Vararuk  A  (2007). Journal of Enterprise Information Management.

[6] SG Jacob, RG Ramani (2012). International Journal of Applied Information Systems.

[7] Fayyad  U (1996). Communications of the ACM.

